# Kaleidoscopic Hues of Antiepileptics in Pediatric Population: Comparison of Prevailing Antiepileptics

**DOI:** 10.7759/cureus.39173

**Published:** 2023-05-18

**Authors:** Abhisek Rout, Chaitali Pattanayak, Reshmi Mishra, Jyoti Prakash Sahoo, Mangala C Das

**Affiliations:** 1 Pharmacology, Kalinga Institute of Medical Sciences, Bhubaneswar, IND; 2 Pharmacology and Therapeutics, Kalinga Institute of Medical Sciences, Bhubaneswar, IND; 3 Pediatrics, Kalinga Institute of Medical Sciences, KIIT (Kalinga Institute of Industrial Technology) University, Bhubaneswar, IND

**Keywords:** newer antiepileptics, heath related quality of life, univariate analysis, epilepsy disorders, seizure free status, pediatric seizure

## Abstract

Background and objectives: Currently, we have a shortage of comprehensive information about newer antiepileptic drugs (AEDs) in the pediatric population. This might explain the discrepancies among pediatricians’ preferences in this regard. Therefore, it is crucial to study the multifaceted impacts of these drugs on children. The endpoints of our study were non-AED predictors of the requirement of combination therapy for seizure management, seizure-free period >6 months and >12 months, change in Quality of Life in Childhood Epilepsy Questionnaire - 55 (QOLCE-55), and incidence of adverse events.

Methods: This prospective, observational study was conducted in KIMS, Bhubaneswar, India, from January 2021 to November 2022. Children of 2-12 years of age were treated with monotherapy of either newer antiepileptics, e.g., levetiracetam, topiramate, and oxcarbazepine or older antiepileptics, e.g., valproic acid, phenytoin, phenobarbital, and carbamazepine. Univariate and multivariate analyses were performed for the assessment of predictors. We used R software (version 4.1.1) for data analysis.

Results: One hundred and ninety-eight (91.7%) of 216 enrolled participants completed this study. The mean age of the study population was 5.2 years and 117 (59%) of them were males. The univariate analysis showed that male gender, low birth weight, preterm birth, assisted vaginal delivery and site-specific epilepsy, and maternal history of epilepsy were significant predictors of combination therapy and reduced seizure-free period. There was a non-significant difference regarding the improvement of QOLCE-55 scores. None of the adverse events were serious.

Conclusions: Perinatal complications and maternal history of epilepsy contribute significantly toward the efficacy of antiepileptics. However, multivariate analysis did not yield statistically significant results.

## Introduction

An assortment of eclectic, multifaceted ailments known as epilepsies affect people physically, psychologically, and socially [1]. Children are generally not enrolled in clinical trials and are given medications envisioned for adults owing to various constraints [2]. Children are not miniature adults. Incidences of epilepsies among children, who are currently in greater numbers worldwide, account for a sizable portion of the total [3,4]. In 2017, 292 million people under the age of 12 had epilepsy worldwide [5]. In 2017, its annual incidence was 58 per every 100,000 children in the Indian subcontinent [6]. As a result, the computation of efficacy data from adult antiepileptic drug (AED) clinical trials has been put forward as a prediction technique to gauge efficacy in children and as an alternative to randomized trials in children [7,8].

Coupled with the pharmacodynamic sensitivity of the AEDs, children's physical and physiological characteristics alter the therapeutic window and response toward various seizure types. Additionally, concerns for parents include polypharmacy, the duration between seizures, quality of life, and AED side effects, all of which cause mental turmoil. Wherefore, there remains a constant need for novel drugs to tackle these issues [2,9,10]. We mapped this study out to evaluate the efficacy, safety, and quality of life of antiepileptics in the pediatric population.

## Materials and methods

This prospective, observational study was conducted from 4th January 2021 to 10th November 2022, in the departments of Pharmacology and Pediatrics, KIMS, Bhubaneswar, India. We obtained approval (KIIT/KIMS/IEC/500/2020, dated 03.11.2020) from the Institutional Ethics Committee, KIMS, Bhubaneswar, before study initiation. Consent from either parent was obtained prior to their child’s enrolment. Both female and male children of 2-12 years of age with a diagnosis of generalized, partial, absence, atonic, or myoclonic epilepsy and managed by a single AED were included. The children diagnosed with complex partial seizure, febrile seizure, status epilepticus, unclear seizure types, and those under multiple AEDs were excluded from the study. This study was prospectively registered with the Clinical Trial Registry, India (CTRI/2020/12/030147). The primary objective was non-AED predictors of the requirement of combination therapy for the management of seizures. The secondary objectives of the study were non-AED predictors of seizure-free period >6 months and >12 months, change in the Quality of Life in Childhood Epilepsy Questionnaire - 55 (QOLCE-55), and incidence of adverse events.

We examined outpatient case sheets of pediatric patients with epilepsy during the above-mentioned period. We reviewed the patient demographics, perinatal and medical history, clinical tolerability, comorbidities, and concomitant medications. The common antiepileptics prescribed in our institution were valproic acid (VPA), carbamazepine (CBZ), phenytoin (PHE), phenobarbital (PBT), levetiracetam (LEV), topiramate (TPM), and oxcarbazepine (OXC). We assessed all eligible participants on the monotherapy of these drugs. All parents were asked to bring their children participating in the study for follow-up visits at monthly intervals for 18-24 months. Seizures not managed with monotherapy of AEDs were dealt with combination of other AEDs. The QOLCE-55 [11] scores were recorded at the baseline visit and after six months of the intervention.

We implemented convenience sampling for this study. The univariate and multivariate analyses were performed for the non-AED predictors of the requirement of combination therapy and seizure-free periods. The variables used for such analyses were age, gender, birth weight, preterm birth, mode of delivery, history of seizure in the first month of life, congenital brain anomalies, brain infections like encephalitis, meningitis, brain tumors, cerebral palsy, developmental disabilities, head injury, site-specific epilepsy, autism-specific disorder, history of post-traumatic seizures, maternal history of epilepsy, and use of psychoactive substances during pregnancy. The intergroup analysis for change in QOLCE-55 scores was done with a one-way analysis of variance (ANOVA). The chi-square (χ^2^) test was used to analyze the adverse events in the study participants. The statistical significance level was set at 0.05. The "R" software (version 4.1.1) [12] was used for the data analysis and generation of the plots.

## Results

We screened 372 children (aged 2-12 years) with epilepsy for this study. One hundred and forty patients were excluded because of their seizure types, parents of 16 children did not give their consent for participation, and the rest 216 were enrolled. Eighteen patients were lost to follow-up. Hence, 198 participants completed the study and were included in the final analysis. The baseline demographic and clinical parameters of the study population are shown in Table 1. Of the participants, 117 (59%) were males. The most common seizure type was generalized seizure (128, 65%) followed by partial (29, 15%), atonic (21, 10%), absence (16, 8%), and myoclonic seizure (4, 2%). The most common drug prescribed for these seizure disorders was LEV (83), VPA (45), PHE (32), CBZ (11), OXC (10), PBT (9), and TPM (8).

**Table 1:** The demographics of the study population by antiepileptic drugs AED: antiepileptics drugs; CBZ: carbamazepine; LEV: levetiracetam; OXC: oxcarbazepine; PBT: phenobarbital; PHE: phenytoin; TPM: topiramate; VPA: valproic acid.

AED	n	Males, n (%)	Mean age (years)	Epilepsy type, n (%)					Mean weight (kg)	Maximum dose (mg/day)
				Generalized	Partial	Absence	Atonic	Myoclonic		
VPA	45	28 (62%)	5.3	28 (62%)	5 (11%)	3 (7%)	5 (11%)	4 (9%)	17.8	300
PHE	32	17 (53%)	4.9	26 (81%)	6 (19%)	0	0	0	18.3	100
PBT	9	3 (33%)	3.9	7 (78%)	2 (22%)	0	0	0	14.6	150
CBZ	11	4 (36%)	5.6	0	1 (7%)	6 (54%)	4 (36%)	0	19.2	600
LEV	83	57 (69%)	5.4	63 (76%)	14 (17%)	0	6 (7%)	0	18.1	300
TPM	8	5 (63%)	4.8	5 (64%)	0	0	3 (36%)	0	17.9	250
OXC	10	3 (30%)	5.1	4 (40%)	1 (10%)	2 (20%)	3 (30%)	0	17.3	600
Total	198	117 (59%)	5.2	128 (65%)	29 (15%)	16 (8%)	21 (10%)	4 (2%)	17.9	

We investigated the predictors of the requirement of combination therapy for the management of epilepsy in the participants. Seventeen variables including age, gender, birth weight, preterm birth, mode of delivery, history of seizure in the first month of life, congenital brain anomalies, brain infections like encephalitis, meningitis, brain tumors, cerebral palsy, developmental disabilities, head injury, site-specific epilepsy, autism-specific disorder, history of post-traumatic seizures, maternal history of epilepsy, and use of psychoactive substances during pregnancy were evaluated as potential predictors of requirement of AED combinations. We performed univariate and multivariate analyses for the non-AED predictors of combination therapy. The findings are illustrated in Table 2. The increased requirement of combined regimens of AEDs for the management of epilepsy in the participants could be attributed to the male gender, children with low birth weight, those born through assisted vaginal delivery like forceps delivery, and those having site-specific epilepsy. However, the multivariate analysis for all 17 predictors were non-significant.

**Table 2:** Non-AED predictors of requirement of combination therapy All 198 patients were assessed for the predictors with 17 variables including age, gender, birth weight, premature birth, perinatal complications, and site-specific epilepsy. Only variables with p <0.05 are shown here. Multivariate analyses of all these variables were non-significant. AEDs: antiepileptic drugs.

Attribute	Number of patients with attribute	Requirement of combination therapy in patients with attribute, n (%)	Requirement of combination therapy in patients without attribute, n (%)	p-Value, univariate	Odds ratio (95% CI), univariate
Male gender	117	15 (12.8%)	9 (11.1%)	0.02	1.15 (1.04-1.28)
Low birth weight	136	22 (16.2%)	7 (11.3%)	0.01	1.43 (1.19-1.67)
Assisted vaginal delivery	71	11 (15.5%)	9 (7.1%)	0.04	2.18 (1.94-2.36)
Site-specific epilepsy	83	17 (20.5%)	18 (15.7%)	0.008	1.31 (1.18-1.54)

We also did the univariate and multivariate analyses for evaluation of the non-AED predictors of seizure-free periods of more than six and 12 months among the study participants. All the above-mentioned variables were evaluated as potential predictors of seizure-free periods. The findings are illustrated in Tables 3, 4. The seizure-free periods could be attributed to low birth weight, preterm birth, and maternal history of epilepsy. However, the multivariate analyses for all the predictors were non-significant.

**Table 3:** Non-AED predictors of seizure-free period >6 months All 198 patients were assessed for the predictors with 17 variables including age, gender, birth weight, premature birth, and perinatal complications. Only variables with p <0.05 are shown here. Multivariate analyses of all these variables were non-significant. AEDs: antiepileptic drugs.

Attribute	Number of patients with attribute	Seizure-free period >6 months in patients with attribute (%)	Seizure-free period >6 months in patients without attribute (%)	p-Value, univariate	Odds ratio (95% CI), univariate
Low birth weight	136	8 (5.9%)	9 (14.5%)	0.03	0.41 (0.32-0.53)
Preterm birth	94	6 (6.4%)	13 (12.5%)	0.03	0.51 (0.37-0.68)
Maternal history of epilepsy	32	5 (15.6%)	33 (19.9%)	0.02	0.78 (0.65-0.94)

**Table 4:** Non-AED predictors of seizure-free period >12 months All 198 patients were assessed for the predictors with 17 variables including age, gender, birth weight, premature birth, and perinatal complications. Only variables with p <0.05 are shown here. Multivariate analyses of all these variables were non-significant. AEDs: antiepileptic drugs.

Attribute	Number of patients with attribute	Seizure-free period >12 months in patients with attribute (%)	Seizure-free period >12 months in patients without attribute (%)	p-Value, univariate	Odds ratio (95% CI), univariate
Low birth weight	136	5 (3.7%)	5 (6.5%)	0.04	0.57 (0.34-0.82)
Preterm birth	94	4 (4.3%)	7 (7.4%)	0.03	0.45 (0.35-0.56)
Maternal history of epilepsy	32	2 (6.3%)	19 (11.4%)	0.04	0.55 (0.37-0.74)

**Figure 1:**
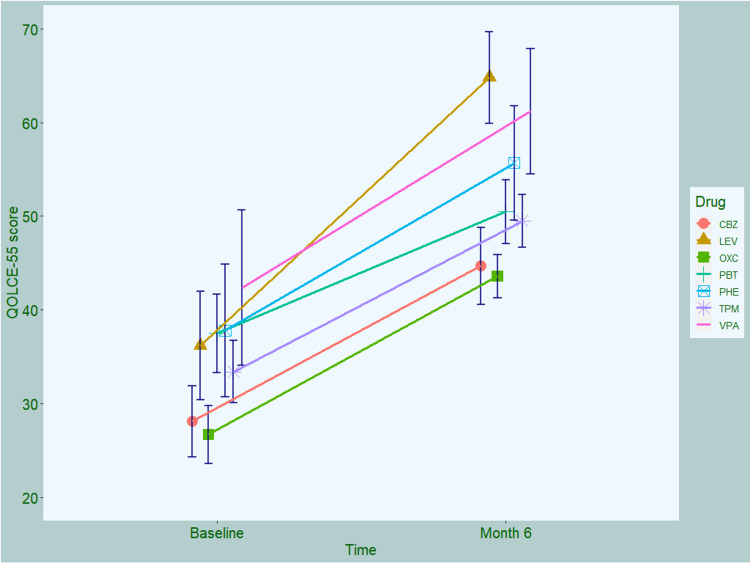
Quality of life of study participants assessed by QOLCE-55 questionnaire The line plots with error bars show the mean values of QOLCE-55 scores. QOLCE-55: Quality of Life in Childhood Epilepsy Questionnaire - 55 (version 1.0); CBZ: carbamazepine; LEV: levetiracetam; OXC: oxcarbazepine; PBT: phenobarbital; PHE: phenytoin; TPM: topiramate; VPA: valproic acid.

The QOLCE-55 scores of the participants are illustrated in Figure 1. The baseline scores [28.1 ± 3.8 (CBZ), 36.2 ± 5.8 (LEV), 26.7 ± 3.1 (OXC), 37.5 ± 4.2 (PBT), 37.8 ± 7.1 (PHE), 33.4 ± 3.3 (TPM), and 42.4 ± 8.3 (VPA), respectively (p = 0.62)] improved after six months of AED interventions [44.7 ± 4.1 (CBZ), 64.8 ± 4.9 (LEV), 43.6 ± 2.3 (OXC), 50.5 ± 3.4 (PBT), 55.7 ± 6.1 (PHE), 49.5 ± 2.8 (TPM), and 61.2 ± 6.7 (VPA), respectively (p = 0.17)]. The intra-group comparison for all the study drugs yielded statistically significant change from the baseline. However, the inter-group comparison did not provide any significant difference among the drugs.

The incidences of adverse events in the study population are shown in Table 5. A total of 298 adverse events were noted in the participants receiving AEDs. The majority of events were seen with LEV (95) followed by VPA (79), PHE (49), PBT (21), OXC (19), CBZ (18), and TPM (17). The most common adverse events were nausea-vomiting (43), increased sleep duration, reduced sleep latency, drowsiness, etc. (43), mild itchy or non-itchy maculo-papular rashes (36), reduced appetite (33), stomach upset (24), lethargy (23), nasal congestion (19), hyperbilirubinemia (18), >10% change from baseline body weight within six months of intervention (18), constipation (16), and mood and behavioral changes (15). None of the events were serious.

**Table 5:** Incidence of adverse events in the study population The p-values were calculated using chi-square (χ^2^) test. CBZ: carbamazepine; LEV: levetiracetam; OXC: oxcarbazepine; PBT: phenobarbital; PHE: phenytoin; TPM: topiramate; VPA: valproic acid; GI: gastrointestinal.

Adverse events	Total (n = 198)	VPA (n = 45)	PHE (n = 32)	PBT (n = 9)	CBZ (n = 11)	LEV (n = 83)	TPM (n = 8)	OXC (n = 10)	p-Value
Total	298	79	49	21	18	95	17	19	0.13
Individual events									
Nausea vomiting	53	9	14	4	3	18	3	2	0.08
Sedation	43	16	6	6	3	2	5	5	0.02
Rash	36	10	11	1	2	11	0	1	0.36
Reduced appetite	33	7	2	0	1	17	3	3	0.003
GI upset	24	5	4	2	3	5	2	3	0.61
Lethargy	23	5	2	3	2	9	0	2	0.41
Nasal congestion	19	2	2	0	1	14	0	0	<0.001
Jaundice	18	13	1	0	1	3	0	0	<0.001
Weight changes	18	8	1	1	0	4	4	0	0.06
Constipation	16	1	5	3	1	4	0	2	0.47
Mood changes	15	3	1	1	1	8	0	1	0.04

## Discussion

This prospective, observational study focused on the efficacy of antiepileptics after six months of intervention in children with epilepsy. All the study participants received the monotherapy of either newer antiepileptics, e.g., LEV, TPM, and OXC, or older antiepileptics, e.g., VPA, PHE, PBT, and CBZ. Whenever required, their doses were gradually increased by the pediatrician. We evaluated the non-AED predictors of the requirement of combination therapy for seizure management, seizure-free period >6 months and >12 months, change in QOLCE-55, and incidence of adverse events. This study ascertained that the need for combined regimens of AEDs was attributed to the male gender, low birth weight, assisted vaginal delivery, and site-specific epilepsy. The seizure-free periods could be attributed to low birth weight, preterm birth, and maternal history of epilepsy. All the study drugs improved the quality of life. However, the intra-group comparison did not show any statistical significance. None of the adverse events were serious.

We found that male children with low birth weight, assisted vaginal delivery, and site-specific epilepsy were less responsive to monotherapy of AEDs. A study by Oliva et al. [13] also suggested that perinatal complications reduce the responsiveness of AEDs leading to the requirement of combination therapy for the same. We observed that children with low birth weight, preterm birth, and maternal history of epilepsy had lower incidences of seizure-free periods than others. A study by Zaccara et al. [14] pointed out that a history of maternal epilepsy and childhood epilepsy increases the seizure frequency, duration, and severity. In our study, the quality of life of the participants was improved with six-month treatment with AEDs. A systematic review by Pellock et al. [15] narrated that the AEDs improve the physical, psychological, and social aspects of quality of life. The antiepileptics in our study had a wide array of adverse effects. Most of them were mild. A study by Beghi E. [16] argued that newer AEDs have better safety profiles than older ones.

Our study was strengthened by univariate and multivariate analyses. However, there were a few limitations to our study as well. First, a smaller sample size, probably because of the multitudinous eligibility criteria of the study and the global pandemic. Secondly, a shorter study duration could have prevented us from long-term efficacy and safety assessments. Thirdly, we could not gather extensive data regarding comorbidities and other concomitant medications. We did not assess the effects of other AEDs.

## Conclusions

We conclude that the male gender, low birth weight, preterm birth, and maternal history of epilepsy contribute significantly toward the heightened necessity of the combination of antiepileptics and reduced seizure-free periods. Despite their side effects, the newer as well as older antiepileptics improve the quality of life. We recommend further studies with a more heterogeneous study population to have one's eye on the long-term effects of these drugs.
